# Taxonomy and distribution of the ant *Cataglyphis setipes* (Hymenoptera: Formicidae)

**DOI:** 10.3897/BDJ.3.e4447

**Published:** 2015-03-27

**Authors:** Aijaz Ahmad Wachkoo, Himender Bharti

**Affiliations:** ‡ University of Kashmir, Srinagar, India; § Punjabi University, Patiala, India

**Keywords:** Formicinae, redescription, ants, distribution, taxonomy

## Abstract

Taxonomy and distribution of the ant species *Cataglyphis setipes* (Forel, 1894) is herewith detailed. *C. setipes* is redescribed, based on workers, queens, and males. Photomontage images of all castes are provided. Information on the distribution and ecology of this species is also given. A key to the Indian species of *Cataglyphis* is presented.

## Introduction

The ant genus *Cataglyphis* Foerster, 1850 is one of the most dominant groups of ants in arid zones of the Old World (Radchenko and Paknia 2010). It is distributed mainly in the Palaearctic region, with several species known from the deserts and semi-deserts of the Afrotropical and Oriental regions (Agosti 1990, Radchenko 1997, Bolton et al. 2007, Radchenko and Paknia 2010). It contains 89 valid species and 20 subspecies in the world fauna (Bolton 2014). This genus is represented by three species in India (Bharti et al. 2014): *C. cugiai* Menozzi, 1939, *C. indica* Pisarski, 1962 and *C. setipes* (Forel, 1894). *C. setipes* is relatively abundant and well represented in collections, but the species has a history of taxonomic confusion. Originally described in 1894, it was then incorrectly treated as a senior synonym of *C. longipedem* (Eichwald, 1841) by Radchenko in 1997 without any argumentation, although latter has the priority (Bolton 2014). However, Radchenko (personal communication, 2015) recognized the error, considers name *C. longipedem* as *incertae sedis* and accordingly revives *C. setipes* from synonymy and treats it as a valid species (Radchenko and Tinaut, in preparation).

*Cataglyphis* male genitalia are highly diverse with many distinct characters helpful in the species delimitation and as a basis for phylogenetic relationships within the genus (Agosti 1990). In recent times, more emphasis has been put on the male caste for identification of species in ant taxonomy due to the discovery of useful characters (Yoshimura and Fisher 2012). Unfortunately it is difficult to collect males since they have restricted periods of emergence throughout the year (Radchenko and Elmes 2010). With the capture of males in more species of ants in the future, it should become easier to classify species based on the characters expressed by males, which in turn will help to decrease the taxonomic impediment that has gripped the Indian ants (Bharti and Wachkoo 2012a, Bharti and Wachkoo 2012b, Bharti and Wachkoo 2012c, Bharti et al. 2014, Wachkoo and Bharti 2014a). Here we are contributing to the study of male ants in this genus by redescribing the male caste of *C. setipes*.

## Materials and methods

The specimens were obtained by visual searching and hand-collecting. The morphological study was conducted with a Nikon SMZ 1500 stereo zoom microscope. For digital images, an Evolution MP digital camera was used on the same microscope with Auto-Montage (Syncroscopy, a division of Synoptics Ltd.) software. The images were processed with Adobe Photoshop CS5. Specimens have been deposited in PUPAC, Punjabi University Patiala Ant Collection. Some worker specimens will be deposited in BMNH, Natural History Museum, London, U.K. and CASC, California Academy of Sciences, San Francisco, United States of America. Morphological terminology for genitalia follow (Boudinot 2013) and for measurements (given in millimeters) and indices found below follow (Bharti and Wachkoo 2014a, Bharti and Wachkoo 2014b, Wachkoo and Bharti 2014b).

HL Maximum length of head in full-face view, measured in straight line from the anterior most point of the clypeal margin to the midpoint of the vertexal margin.

HW Maximum width of head in full-face view (excluding the portion of eyes that protrudes beyond the lateral margins of the head).

EL Maximum length of eye as measured normally in oblique view of the head to show full surface of eye.

SL Maximum length of the scape excluding the basal neck and condyle.

PnW Maximum width of pronotum in dorsal view.

WL Weber’s length measured from the anterior surface of the pronotum proper (excluding the collar) to the posteriormost point of the propodeal lobes.

PL Maximum longitudinal distance in lateral view between the anterior and posterior extensions of the petiolar node, excluding the anterior and posterior condyles.

PW Maximum width of the petiole in dorsal view.

GL Length of the gaster in profile from the anteriormost point of the first gastral segment to the posteriormost point.

CI Cephalic index: HW/HL x 100.

SI Scape index: SL/HW x 100.

REL Relative eye length index: EL/HL x 100.

## Taxon treatments

### Cataglyphis setipes

(Forel, 1894)

Myrmecocystus viaticus r. *setipes*Forel 1894 401 (w.) India (original description). Ruzsky 1902: 9 (q.m.); Imai et al. 1984: 9 (k.). Combination in *Cataglyphis*: Wheeler 1922: 945. Subspecies of *bicolor*: Emery 1906: 58; Emery 1908: 217; Santschi 1929: 49. Raised to species: Bingham 1903: 312; Collingwood 1961b: 289; Collingwood 1961a: 65; Arnol'di 1964: 1804; Arnol'di and Dlussky 1978: 554. Senior synonym of *turcomanica*: Dlussky et al. 1990: 155; Radchenko 1997: 435; of *dschambulica* and material of the unavailable name *setipesdesertorum*. Radchenko 1997: 435 places *longipedem* incorrectly as a junior synonym of *setipes*, but *longipedem* has priority. Revived from synonymy: Radchenko and Tinaut (in preparation).

#### Materials

- **Type status:** Syntype. **Occurrence:** recordedBy: A. Forel; individualCount: 1; sex: worker; **Location:** country: India; locality: Nusseerabad, Rajpootana; **Record Level:** institutionCode: MHNG, Geneva, Switzerland; collectionCode: CASENT0249882
- **Type status:** Other material. **Occurrence:** recordedBy: Aijaz A. Wachkoo; individualCount: 3; sex: workers; **Location:** country: India; stateProvince: Himachal Pradesh; locality: Andretta; verbatimElevation: 930 m; verbatimCoordinates: 30°20.56'N 119°26.03'E; verbatimLatitude: 32.0744°N; verbatimLongitude: 76.5856°E; **Event:** eventDate: Jun-21-2010; **Record Level:** institutionCode: PUPAC
- **Type status:** Other material. **Occurrence:** recordedBy: Aijaz A. Wachkoo; individualCount: 2; sex: workers; **Location:** country: India; stateProvince: Himachal Pradesh; locality: Jogi Panga; verbatimElevation: 600 m; verbatimCoordinates: 30°20.56'N 119°26.03'E; verbatimLatitude: 31.3229°N; verbatimLongitude: 76.1858°E; **Event:** eventDate: Sep-09-2008; **Record Level:** institutionCode: PUPAC
- **Type status:** Other material. **Occurrence:** recordedBy: Aijaz A. Wachkoo; individualCount: 9; sex: workers; **Location:** country: India; stateProvince: Himachal Pradesh; locality: Khatiar; verbatimElevation: 450 m; verbatimLatitude: 31.5810°N; verbatimLongitude: 75.5516°E; **Event:** eventDate: Jun-03-2009; **Record Level:** institutionCode: PUPAC
- **Type status:** Other material. **Occurrence:** recordedBy: Aijaz A. Wachkoo; individualCount: 21; sex: workers; **Location:** country: India; stateProvince: Himachal Pradesh; locality: Khatiar; verbatimElevation: 450 m; verbatimCoordinates: 30°20.56'N 119°26.03'E; verbatimLatitude: 31.5810°N; verbatimLongitude: 75.5516°E; **Event:** eventDate: Oct-11-2009; **Record Level:** institutionCode: PUPAC
- **Type status:** Other material. **Occurrence:** recordedBy: Aijaz A. Wachkoo; individualCount: 1; sex: queen; **Location:** country: India; stateProvince: Himachal Pradesh; locality: Khatiar; verbatimElevation: 450 m; verbatimLatitude: 31.5810°N; verbatimLongitude: 75.5516°E; **Event:** eventDate: Oct-11-2009; **Record Level:** institutionCode: PUPAC
- **Type status:** Other material. **Occurrence:** recordedBy: Aijaz A. Wachkoo; individualCount: 6; sex: males; **Location:** country: India; stateProvince: Himachal Pradesh; locality: Khatiar; verbatimElevation: 450 m; verbatimLatitude: 31.5810°N; verbatimLongitude: 75.5516°E; **Event:** eventDate: Oct-11-2009; **Record Level:** institutionCode: PUPAC
- **Type status:** Other material. **Occurrence:** recordedBy: Aijaz A. Wachkoo; individualCount: 5; sex: workers; **Location:** country: India; stateProvince: Himachal Pradesh; locality: Kotla; verbatimElevation: 500 m; verbatimLatitude: 31.8821°N; verbatimLongitude: 75.9963°E; **Event:** eventDate: Oct-13-2008; **Record Level:** institutionCode: PUPAC
- **Type status:** Other material. **Occurrence:** recordedBy: Aijaz A. Wachkoo; individualCount: 1; sex: worker; **Location:** country: India; stateProvince: Himachal Pradesh; locality: Nahan; verbatimElevation: 760 m; verbatimLatitude: 30.5596°N; verbatimLongitude: 77.2960°E; **Event:** eventDate: Aug-23-2009; **Record Level:** institutionCode: PUPAC
- **Type status:** Other material. **Occurrence:** recordedBy: Aijaz A. Wachkoo; individualCount: 2; sex: workers; **Location:** country: India; stateProvince: Himachal Pradesh; locality: Poanta Sahib; verbatimElevation: 420 m; verbatimLatitude: 30.4384°N; verbatimLongitude: 77.6239°E; **Event:** eventDate: Aug-19-2009; **Record Level:** institutionCode: PUPAC
- **Type status:** Other material. **Occurrence:** recordedBy: Aijaz A. Wachkoo; individualCount: 3; sex: workers; **Location:** country: India; stateProvince: Himachal Pradesh; locality: Renuka; verbatimElevation: 600 m; verbatimLatitude: 30.6083°N; verbatimLongitude: 77.4615°E; **Event:** eventDate: Aug-26-2009; **Record Level:** institutionCode: PUPAC
- **Type status:** Other material. **Occurrence:** recordedBy: Aijaz A. Wachkoo; individualCount: 1; sex: worker; **Location:** country: India; stateProvince: Himachal Pradesh; locality: Terrace; verbatimElevation: 430 m; verbatimLatitude: 31.9234°N; verbatimLongitude: 75.9294°E; **Event:** eventDate: May-25-2009; **Record Level:** institutionCode: PUPAC
- **Type status:** Other material. **Occurrence:** recordedBy: Aijaz A. Wachkoo; individualCount: 3; sex: workers; **Location:** country: India; stateProvince: Jammu and Kashmir; locality: Manda; verbatimElevation: 500 m; verbatimLatitude: 32.7496°N; verbatimLongitude: 74.8673°E; **Event:** eventDate: Aug-04-2010; **Record Level:** institutionCode: PUPAC
- **Type status:** Other material. **Occurrence:** recordedBy: Aijaz A. Wachkoo; individualCount: 1; sex: worker; **Location:** country: India; stateProvince: Jammu and Kashmir; locality: Mansar; verbatimElevation: 690 m; verbatimLatitude: 32.6979°N; verbatimLongitude: 75.1489°E; **Event:** eventDate: Aug-03-2010; **Record Level:** institutionCode: PUPAC
- **Type status:** Other material. **Occurrence:** recordedBy: Aijaz A. Wachkoo; individualCount: 3; sex: workers; **Location:** country: India; stateProvince: Jammu and Kashmir; locality: Surinsar; verbatimElevation: 700 m; verbatimLatitude: 32.7009°N; verbatimLongitude: 75.1512°E; **Event:** eventDate: Jul-14-2009; **Record Level:** institutionCode: PUPAC
- **Type status:** Other material. **Occurrence:** recordedBy: Aijaz A. Wachkoo; individualCount: 3; sex: workers; **Location:** country: India; stateProvince: Uttarakhand; locality: Ranger’s College, Dehradun; verbatimElevation: 660 m; verbatimLatitude: 30.1921°N; verbatimLongitude: 78.2400°E; **Event:** eventDate: May-27-2010; **Record Level:** institutionCode: PUPAC
- **Type status:** Other material. **Occurrence:** recordedBy: Aijaz A. Wachkoo; individualCount: 1; sex: worker; **Location:** country: India; stateProvince: Uttarakhand; locality: Selaqui, Dehradun; verbatimElevation: 670 m; verbatimLatitude: 30.3720°N; verbatimLongitude: 77.8605°E; **Event:** eventDate: Aug-08-2009; **Record Level:** institutionCode: PUPAC

#### Description

**Worker** (Fig. 1).

**Worker measurements:** HL 1.54-2.97; HW 1.32-3.08; EL 0.42-0.77; SL 1.90-3.24; PnW 0.99-1.99; WL 2.55-4.90; PL 0.37-0.74; PW 0.31-0.69; GL 1.80-4.22 mm. Indices: CI 86-104; SI 105-144; REL 25-28 (n=25).

Head with almost parallel sides and gradually convex posterior margin, wider than long in major worker and longer than wide in minor worker; clypeus carinate in major worker and subcarinate in minor worker; anterior clypeal margin convex, medially shallowly concave to transverse; eyes prominent, situated distinctly above the midlength of lateral head margins; 3 small ocelli present; antennae 12-segmented, scapes long, distinctly longer than head length, surpass posterior margin by about one-third of their length in major worker and by half of their length in minor worker; mandibles with 5 to 7 teeth, if more than 5 teeth present, then the third tooth counting from the apex is larger and longer than the fourth; fourth tooth smaller in size than basal two in 6 -toothed specimens; fourth and fifth smaller than basal two in 7-toothed specimens. Maxillae with long hairs; basal segment of maxillae flat.

Mesosoma typical for this genus; pronotum convex; propodeum low, gradually arched, its dorsal surface distinctly longer than posterior one; propodeal spiracles distinctly slit-like, and very long; petiole obviously nodiform, with rounded node dorsum; long legs.

Surface of whole body covered with dense microreticulation, appears dull, although not strongly matt; mandibles striate with few scattered punctures.

Body with sparse standing pilosity, denser on head and the underside of gaster; pubescence minute and fine, relatively denser on mesosoma with a silvery glint; antennal funiculus with fine, short appressed to decumbent pubescence, scapes with suberect hairs; legs covered with dense macrosetae.

Head, mesosoma and node of petiole dark red; gaster black; legs a shade darker than mesosoma, almost black.

**Queen** (Fig. 2).

**Queen measurements:** HL 2.79; HW 2.85; EL 0.77; SL 2.55; WL 4.56; PL 0.67; PW 0.96; GL 5.27 mm. Indices: CI 102; SI 89; REL 28 (n=1).

As in major worker, with modifications expected for caste and the following differences: head narrower and scapes shorter than in major worker, surpassing posterior margin by three-tenths their length; clypeus subcarinate; mesosoma enlarged, mesonotum not constricted; petiole compressed anterolaterally, narrower in profile but wider in dorsal view than in major worker; mesepimeron with a posterodorsal (epimeral) lobe that covers mesothoracic spiracle and forms a seemingly isolated plate.

**Male** (Fig. 3).

**Male measurements:** HL 1.80-2.04; HW 1.86-1.94; EL 0.63-0.76; SL 2.61-2.91; WL 3.98-4.28; PL 0.46-0.50; PW 0.75-0.94; GL 4.46-4.96 mm. Indices: CI 95-103; SI 140-150; REL 35-37 (n=5).

Head subquadrate about as long as wide; eyes subglobulose, convex, large and bulging, breaking head outline in full-face view; 3prominent ocelli present; antennae 13-segmented, filiform, scapes long, surpass posterior margin of head by more than half their length; clypeus subcarinate in some specimens with round anterolateral corners; mandibles slender, curved and strap-like, apical tooth simple, acute, remainder without any dentition in some specimens and with well differentiated apical and basal tooth in some specimens.

Notauli absent; parapsidal lines prominent, diverging anteriorly; mesepimeron with a posterodorsal (epimeral) lobe that covers mesothoracic spiracle and forms a seemingly isolated plate; jugal lobe of hind wing absent; dorsal margin of petiole, in anterior view, shallowly concave to broadly round; propodeal declivity broadly rounded; propodeal spiracle elongate, slit shaped.

Basimeres large, broad at the base and tapering to a blunt point; in dorsal view, telomeres elongate anteroposteriorly, oval and rounded apically in lateral view covered by scrobiculae; both the basimeres and telomeres are setose; basimedialtelomeral process with flat broad parallel base, apex roughly dumbbell shaped, about one third the length of the telomere; cuspides small, triangular,with peg-like teeth on medial face, bent toward digiti; digiti smooth, much longer than cuspides, about 2 times the length of cuspides and usually bent towards each other apically; in lateral view digiti falcate and gently downcurved; penisvalva projecting with apices of each penisvalva directed posterolaterally. Subgenital plate concave and bilobed posterolaterally, with short triangular process in the centre of posterior margin.

Body covered with relatively more dense erect hairs especially on underside of gaster and parameres than in other conspecific castes, in addition to normal pubescence.

Head, mesosoma and petiole black, gaster light brown; sculpture as in worker caste.

#### Diagnosis

This species most resembles *C. indica* from which it separates by lighter body colour; dense setae on tibiae and rounded propodeum whilst latter is characterized by darker body colour, sparse setae on tibiae and much angular propodeum. However, the workers are rather variable in the characters used to differentiate *C. setipes* and *C. indica* and therefore, discovery of males of *C. indica* in future, may prove pivotal in taxonomic decision regarding its validity based on the characters expressed by males.

#### Distribution

This is one of the most conspicuous ant species found commonly in arid and semiarid zones of Central and South Asia.

#### Ecology

This species inhabits subtropical areas and is relatively easy to find because they preferentially occupy open habitats. These ants have been observed to form permanent nests in dry soil; nests can be easily located in bare ground and along roadside. Workers of this species usually forage individually and raise gaster in locomotion.

## Identification Keys

### Key to Indian species of *Cataglyphis* (workers)

**Table:** 

1	Petiolar node squamiform, compressed longitudinally, its dorsal margin narrow (Fig. 4)	*C. cugiai* Menozzi, 1939
–	Petiolar node nodiform, not compressed longitudinally, dorsum broader, subquadrate (Fig. 1​)	2
2	Propodeum angular, body brown	*C. indica* Pisarski, 1961
–	Propodeum rounded, body reddish brown	*C. setipes* (Forel, 1894)

## Supplementary Material

XML Treatment for Cataglyphis setipes

## Figures and Tables

**Figure 1a.**
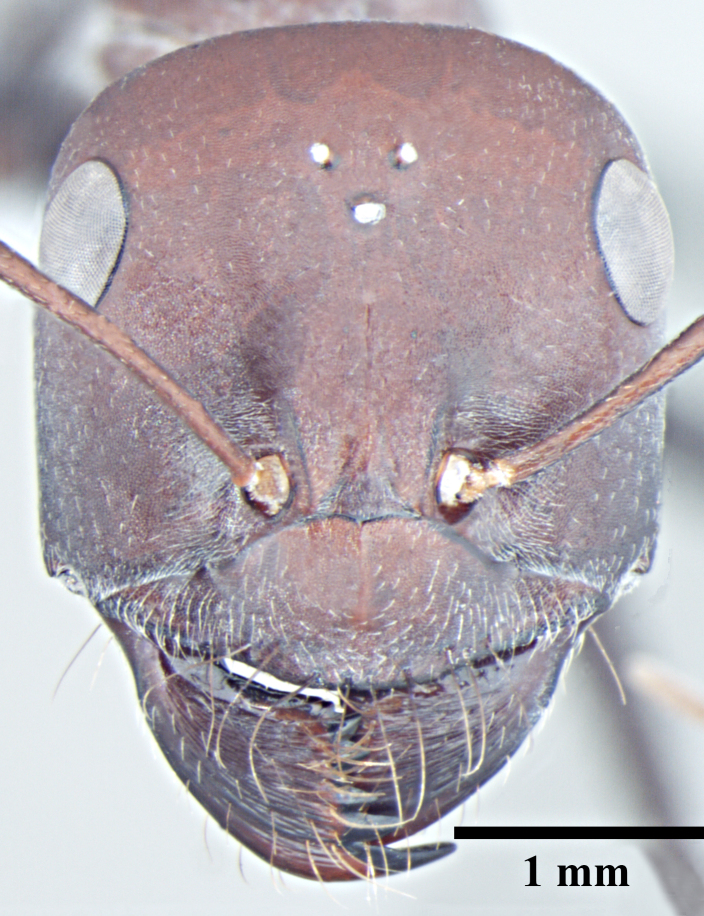
Major worker head, full face view

**Figure 1b.**
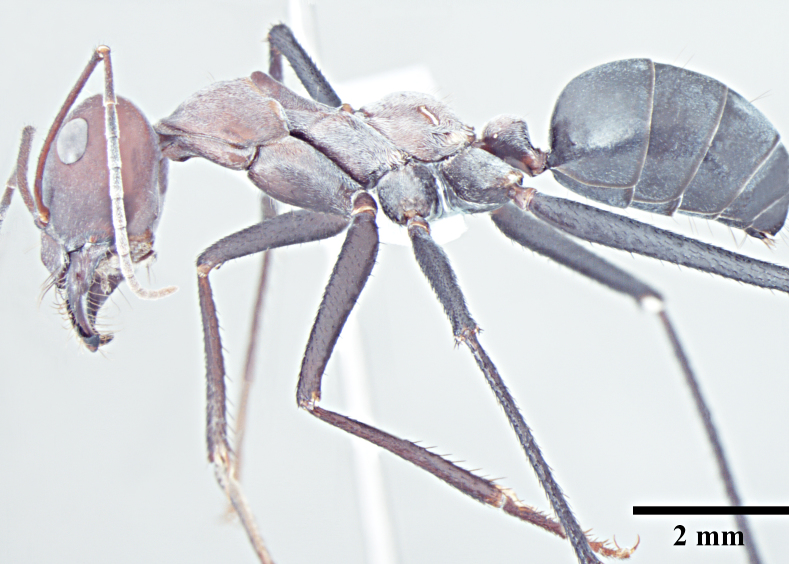
Major worker body, lateral view

**Figure 1c.**
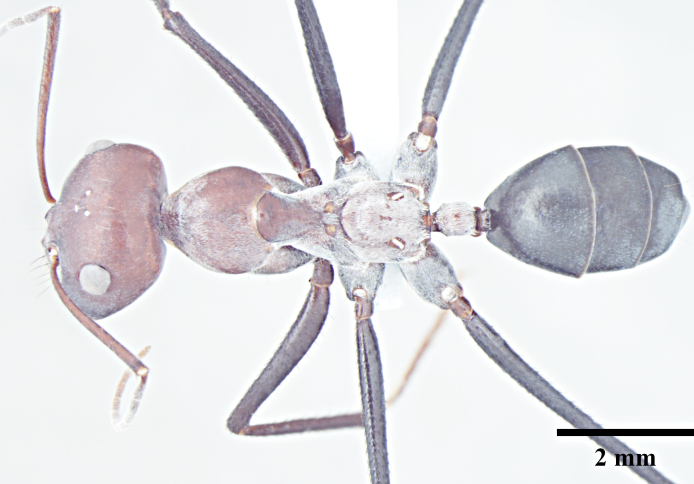
Major worker body, dorsal view

**Figure 1d.**
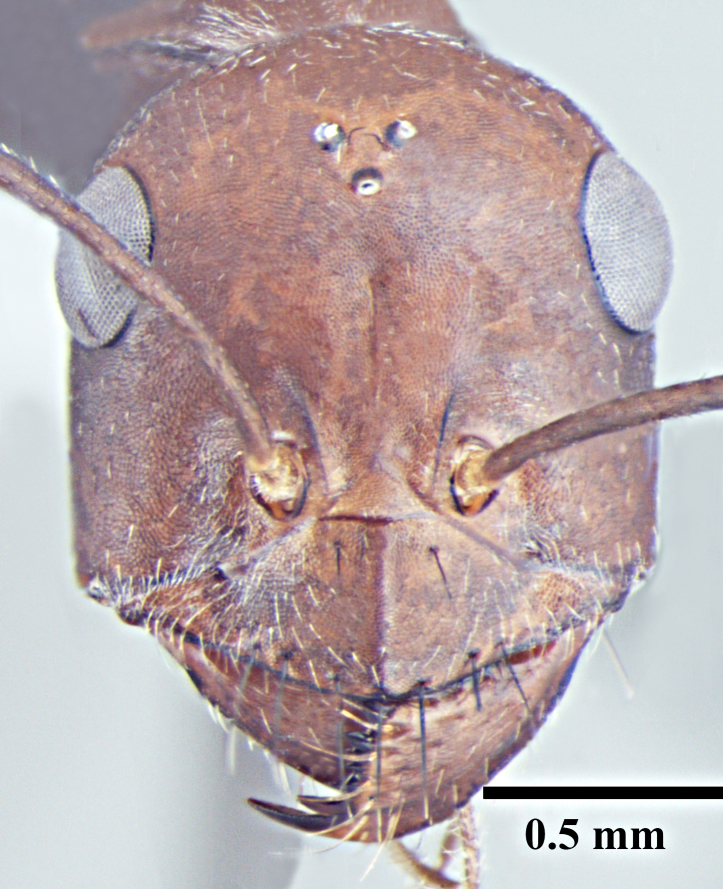
Minor worker head, full face view

**Figure 1e.**
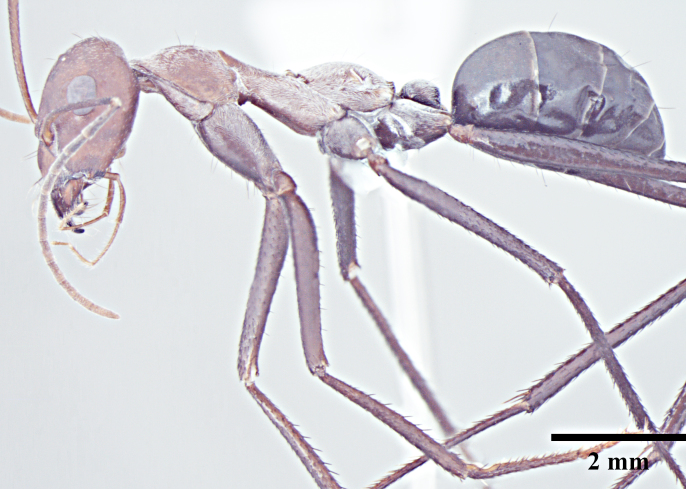
Minor worker body, lateral view

**Figure 1f.**
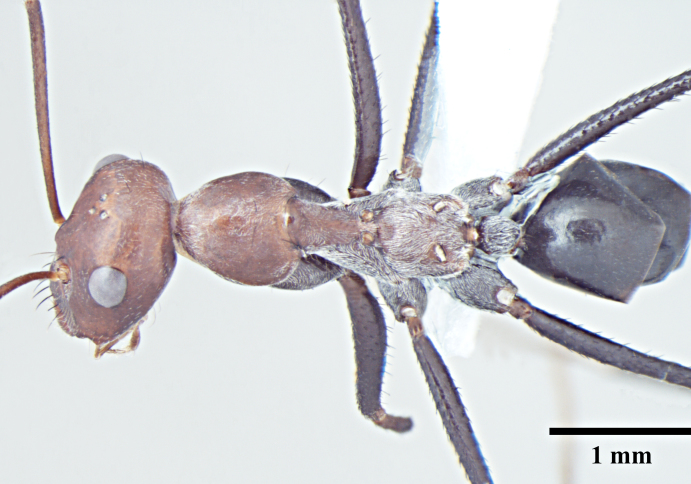
Minor worker body, dorsal view

**Figure 2a.**
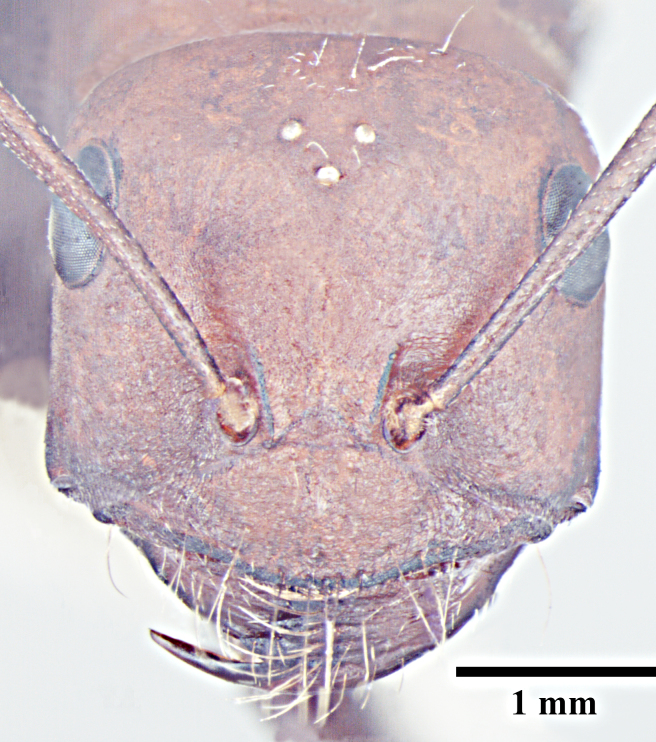
Queen head, full face view

**Figure 2b.**
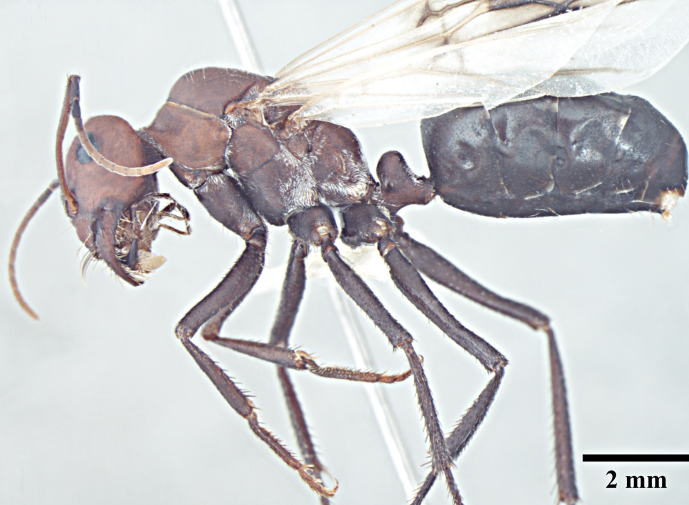
Queen body, lateral view

**Figure 2c.**
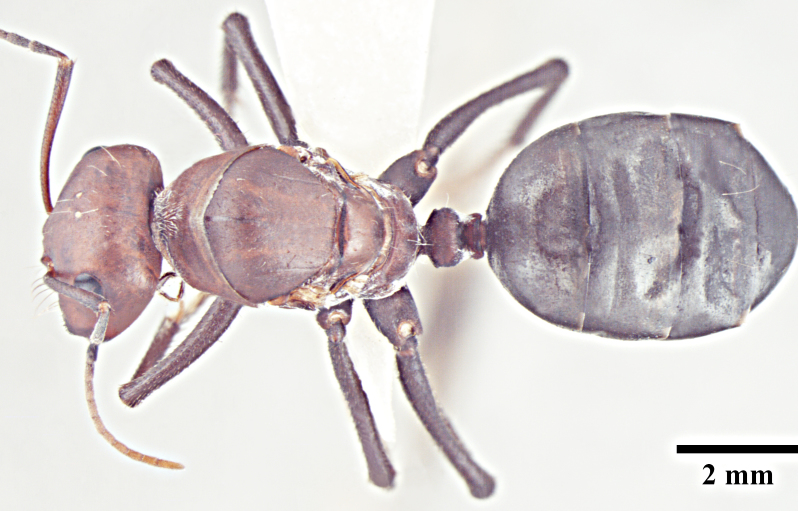
Queen body, dorsal view

**Figure 3a.**
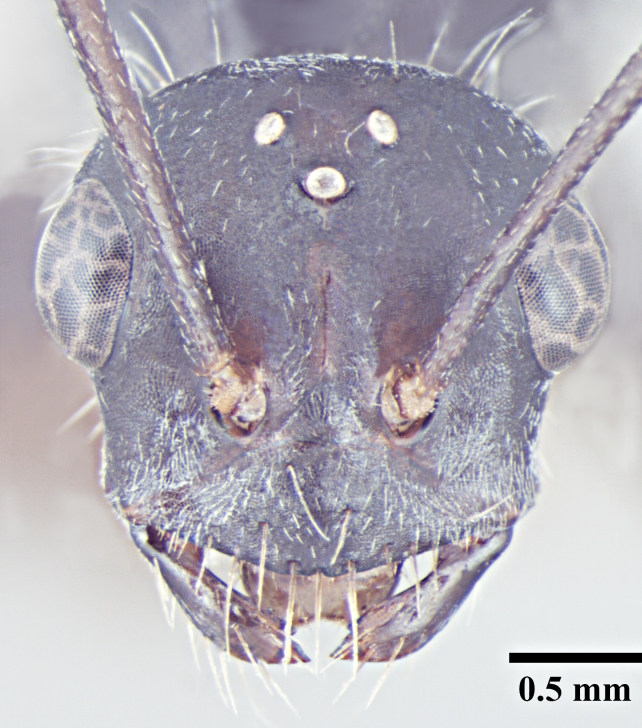
Male head, full face view

**Figure 3b.**
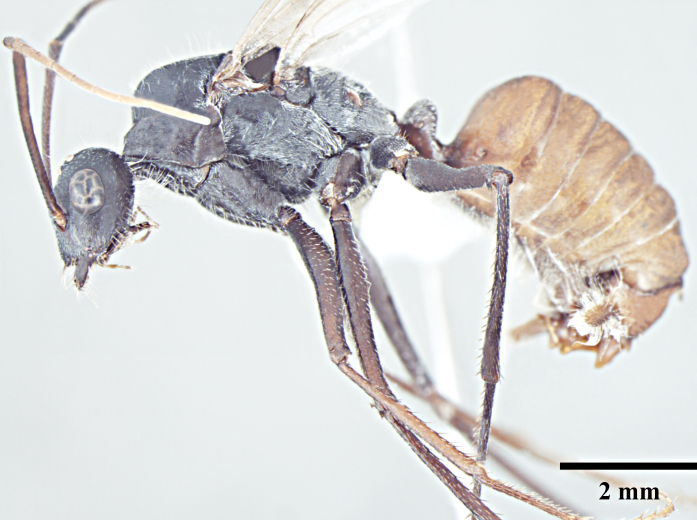
Male body, lateral view

**Figure 3c.**
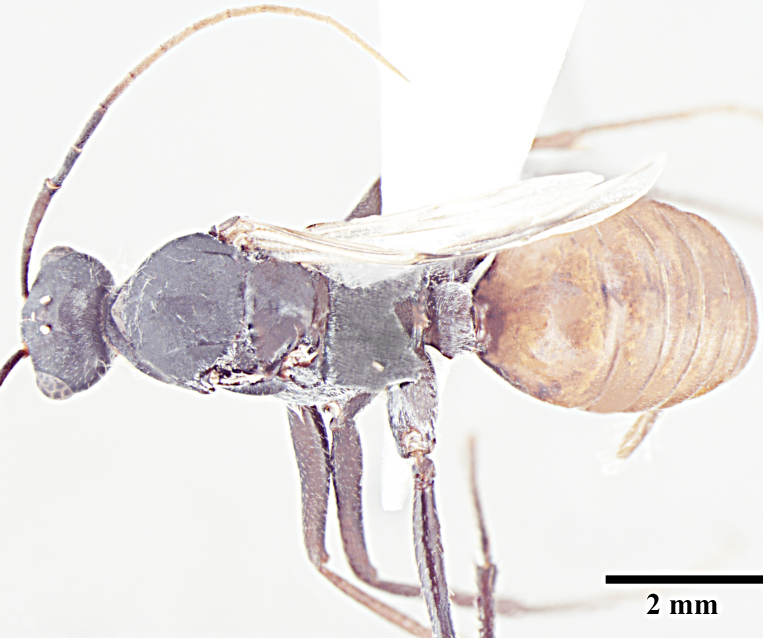
Male body, dorsal view

**Figure 3d.**
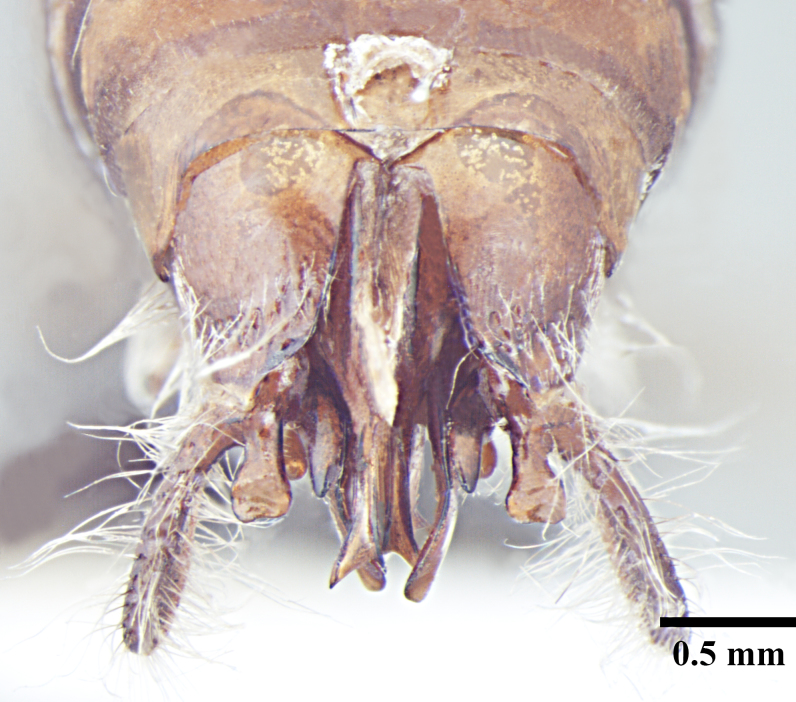
Male, genitalia

**Figure 4a.**
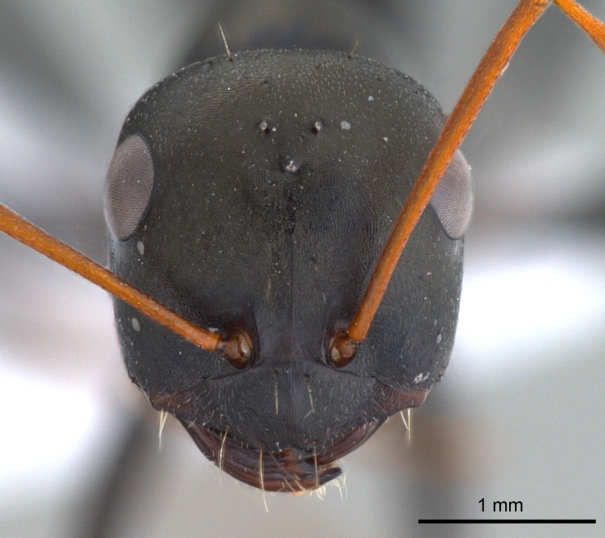
Worker head, full face view

**Figure 4b.**
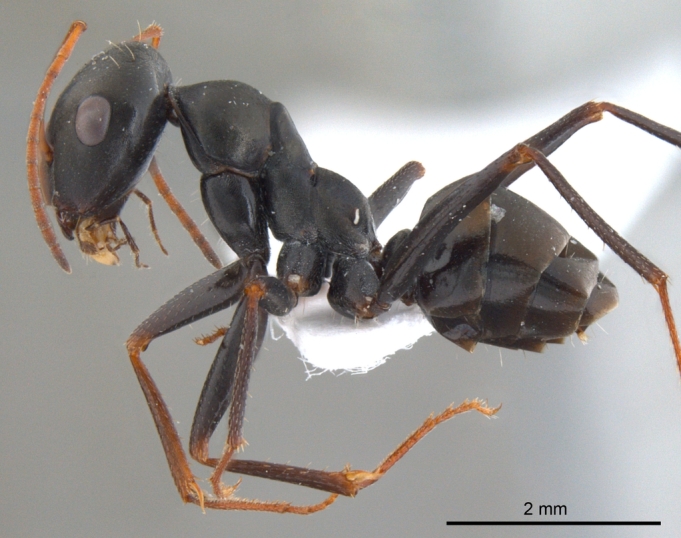
Worker body, lateral view

**Figure 4c.**
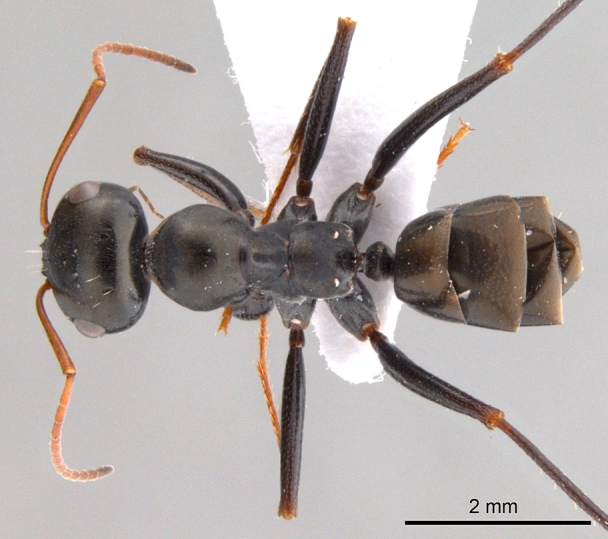
Worker body, dorsal view
